# Videomicroscopic investigation of the microcirculation requires uniform definitions

**DOI:** 10.14814/phy2.13303

**Published:** 2017-06-11

**Authors:** Özge Erdem, Jan Willem Kuiper, Can Ince, Dick Tibboel

**Affiliations:** ^1^ Intensive Care and Department of Pediatric SurgeryErasmus Medical Center – Sophia Children's HospitalRotterdamthe Netherlands; ^2^ Department of Translational PhysiologyAcademic Medical CenterAmsterdamthe Netherlands; ^3^ Department of Intensive CareErasmus Medical CenterRotterdamthe Netherlands

*On article*: Wright IM, Latter JL, Dyson RM, Levi CR, Clifton VL. Videomicroscopy as a tool for investigation of the microcirculation in the newborn. Physiol Rep. 2016;4(19):e12941

Dear Editor, We would like to compliment Wright et al. (2016) for their extensive data which can aid ongoing research in a very important field and like to share a number of important considerations.

Multiple generations of video microscopy techniques are available for the assessment of the microcirculation. The first video microscopy technique is based on *orthogonal polarization spectral* (OPS) imaging (Groner et al. 1999). The reported Microscan (Microvision Medical) is a second generation video microscopy which operates with *sidestream dark field* (SDF) imaging (Goedhart et al. 2007). This technique is superior to OPS imaging in capillary contrast and quality (Groner et al. 1999). However, unlike stated in the introduction, SDF imaging neither uses polarized light nor is it fitted with a polarized filter, this is the case in OPS imaging which is another technology. We are confused therefore which technique is used for this study of Wright, because both generations are referred to in the third paragraph of the introduction. It is also worth mentioning that, recently, a third‐generation technique based on *incident dark field illumination* (IDF) became available (Aykut et al. 2015). This technique provides better image quality and detects more capillaries compared with SDF imaging and has already been validated for use in adults and neonates (van Elteren et al. 2015). This should be noted in the shortcomings of their study as the capillary density they report with SDF imaging may in fact be much larger.

Secondly, we have some questions about the reported results. It seems arguable that mean values of *vessel perfused length* are higher than mean values of *vessel length*, for example, *large vessel length* (Table 2). It seems unlikely that more vessel length is perfused than the actual length of the vessel?

In the consensus statement for microcirculatory measurements the equation for PVD is formulated as follows: *perfused vessel density* (PVD)(*mm/mm* ^*2*^) = *total vessel density* (TVD)(*mm/mm* ^*2*^) * *proportion perfused vessels* (PPV)(*%*)(De Backer et al. 2007; Massey and Shapiro 2016) Following this equation, PVD cannot take on values higher than values of TVD. This is in contrast to the results for *Other vessels PVD* (Table 2), even considering these are mean values. Also, values of PVD cannot be equal to values of TVD, when the values of PPV are < 100%, see for example the reported *Small Vessels PPV and PVD* in Table 2. Furthermore, it is unlikely to have a mean value of *All vessels PPV* of 100% if the mean value of *Small vessels PPV* is lower than 100%, considering that the calculation of *All vessels PPV* also includes the proportion of perfused small vessels. The observed issues may reflect the use of different definitions for the same variable. Therefore, it would be helpful to know which definitions were used (De Backer et al. 2007; Bezemer et al. 2008).

The reported data could certainly be valuable for future research on the neonatal microcirculation. Hence it is necessary to clearly state which technique is used to assess measurements and to clarify the abovementioned issues in the results.

## Conflict of Interest

Dr. Ince developed SDF imaging and is listed as inventor on related patents commercialized by MicroVision Medical (MVM) under a license from the Academic Medical Center (AMC). He has been a consultant for MVM in the past, but has not been involved with this company for more than five years now and holds no shares. Braedius Medical, a company owned by a relative of dr. Ince, has developed and designed a hand held microscope called CytoCam‐IDF imaging. Dr. Ince has no financial relation with Braedius Medical of any sort, that is, never owned shares, or received consultancy or speaker fees from Braedius Medical.
